# Homosexual Women Have Less Grey Matter in Perirhinal Cortex than Heterosexual Women

**DOI:** 10.1371/journal.pone.0000762

**Published:** 2007-08-22

**Authors:** Jorge Ponseti, Hartwig R. Siebner, Stefan Klöppel, Stephan Wolff, Oliver Granert, Olav Jansen, Hubertus M. Mehdorn, Hartmut A. Bosinski

**Affiliations:** 1 Section of Sexual Medicine, Christian-Albrechts University, Kiel, Germany; 2 Department of Neurology, Christian-Albrechts University, Kiel, Germany; 3 NeuroImage Nord, Hamburg-Kiel-Lübeck, Germany; 4 Wellcome Trust Centre for Neuroimaging, University College London, London, United Kingdom; 5 Department of Neurology, University Freiburg, Freiburg, Germany; 6 Section of Neuroradiology, Department of Neurosurgery, Christian-Albrechts University, Kiel, Germany; 7 Department of Neurosurgery, Christian-Albrechts University, Kiel, Germany; University of Exeter, United Kingdom

## Abstract

Is sexual orientation associated with structural differences in the brain? To address this question, 80 homosexual and heterosexual men and women (16 homosexual men and 15 homosexual women) underwent structural MRI. We used voxel-based morphometry to test for differences in grey matter concentration associated with gender and sexual orientation. Compared with heterosexual women, homosexual women displayed less grey matter bilaterally in the temporo-basal cortex, ventral cerebellum, and left ventral premotor cortex. The relative decrease in grey matter was most prominent in the left perirhinal cortex. The left perirhinal area also showed less grey matter in heterosexual men than in heterosexual women. Thus, in homosexual women, the perirhinal cortex grey matter displayed a more male-like structural pattern. This is in accordance with previous research that revealed signs of sex-atypical prenatal androgenization in homosexual women, but not in homosexual men. The relevance of the perirhinal area for high order multimodal (olfactory and visual) object, social, and sexual processing is discussed.

## Introduction

In humans, structural brain differences between men and women are well known. Total brain size, as well as grey matter (GM) and white matter (WM) size were found to be bigger in men than in women [1]–[7]. On the other hand, women have more GM volume relative to WM volume than men [8] and a higher GM concentration in widespread areas spanning the entire cortical mantle [2], [3], [9], as has been revealed by studies employing voxel based morphology (VBM) [10]. Similarly, the analysis of cortical thickness revealed large areas of increased cortical (GM) thickness in women compared to men [4], [11]. Gender related differences were found to be more pronounced in GM than in WM, particular if GM was calculated in normalized space, usually referred to as GM concentration. However, there is little consensus regarding the localization of the GM areas found to be sex-dimorphic (i.e. showing a gender related difference).

Sexual dimorphism of the human brain has been related to effects of prenatal androgenization [1]. Since sexual orientation (i.e. sexual attraction to members of the opposite-sex or the same-sex) has also been related to prenatal androgenization [12], [13], we were interested if effects of sexual orientation could be found in brain morphology. Previous reports in this regard were rare and ambiguous. To our knowledge studies comparing brain morphology of heterosexual and homosexual women had not yet been published. Microscopic postmortem studies regarding men revealed controversial results: LeVay [14] reported the interstitial nucleus 3 of the human anterior hypothalamus (INAH3) of homosexual men to be more female-like , i.e., of significantly smaller volume and containing less neurons, while Byne et al. [15] found only a non-significant trend for INAH3 to occupy a smaller volume in homosexual than in heterosexual men. Allen and Gorski [16] reported the anterior commissure to be more female-like in homosexual men, a finding that could not be replicated by Lasco et al. [17]. A reported difference in suprachiasmatic nucleus between homosexual and heterosexual men [18] was not replicated by others. A recent VBM study revealed no differences on grey matter volume between heterosexual and homosexual men [19]. However, the authors interpreted this finding as a consequence of small sample size (12 homosexual and 12 heterosexual men).

In an effort to investigate differences in brain morphology between heterosexual and homosexual men and women, we performed a VBM study. We first calculated gender related whole brain effects on GM, WM and cerebrospinal fluid (CSF) to check the comparability of our sample. Since gender effects were found to be pronounced when GM images were calculated with unmodulated brain images, we used unmodulated images to test for differences in the regional probability to belong to GM (concentration) [20]. We wanted to know (a) if homosexual men and women differ from heterosexual men and women in terms of GM, i.e. if we could detect areas of increased GM in homosexual men and areas of decreased GM in homosexual women; (b) if these divergences occur particularly in those brain areas that we could demonstrate to be sex-dimorphic. The latter would support the concept that a gender-typical differentiation of the given brain areas is attenuated in homosexual individuals.

## Materials and Methods

### Subjects

Twenty-five heterosexual women (mean age: 24.9±3.3 ys.), 24 heterosexual men (mean age: 25.3±3.2 ys.), 16 homosexual men (mean age: 27.3±3.7 ys.), and 15 homosexual women (mean age: 24.9±5.8 ys.) participated in the study. A subset had been involved in a previous functional MRI study [21]. A two-way ANOVA revealed neither a significant age-difference between males and females (*F*_1_ = 2.5, *P* = 0.12), or between homosexual and heterosexual probands (*F*_1_ = 1.2, *P* = 0.27), nor an interaction between the two grouping factors sex of the participant and sexual orientation of the participant (*F*_1_ = 1.3, *P* = 0.26). All participants gave their written informed consent before they participated in the experiment. Subjects were pre-screened by means of an inventory to verify that they were right-handed [22], and mentally not distressed [23]. In a structured interview, we verified that participants were heterosexual respectively homosexual (i.e., Kinsey rating of fantasy and behaviour of 0 and 1 or 5 and 6, respectively) [24], and had no history of substance abuse, sexual dysfunction, gender identity disorder, paraphilia or sexual offences. The study was approved by the Ethics committee of the Medical Faculty of the Christian-Albrechts-University. Subjects were enrolled by advertisements on campus and in a homosexual self-support group, asking for participation in a study on sexual brain research.

### Data acquisition, image processing, and statistical analysis

MRI scanning was performed on a 1.5-Tesla Philips Intera scanner using a quadrature head coil. A structural MRI was acquired on each participant using a 3D T1 weighted sequence (TR 7.9, TE 3.7 ms, flip angle 8°, FOV 220×132×178 mm^3^, 256×256 matrix, 1.2 mm slice-thickness) yielding 110 sagittal slices.

Data pre-processing and statistical analysis was performed using SPM2 software (http://www.fil.ion.ucl.ac.uk/spm/) running under Matlab (MathWorks, Natick, MA).

In brief, VBM comprises first, spatial normalization of all images to a standardized anatomical space to allow spatial averaging, second, segmentation of images into the three major tissue compartments (GM, WM, and CSF) and third, comparison of local GM concentration across the whole brain [10]. We used a so-called optimized VBM protocol [20] using a study-specific template based on the T1-image from every subject in the study. Resulting GM segments were averaged and smoothed with a 12 mm Gaussian kernel.

Global effects of gender were tested on each tissue type with mean voxel values as dependent variable using two-way ANOVAs (with gender and sexual orientation as grouping factors). In addition, post hoc t-test within gender comparisons were performed for exploratory purpose. Regional GM concentration was compared using a two sample t-tests, while controlling for global GM mean voxel values to remove variance due to differences in brain tissue size. In this way we performed comparisons between male and female brains, and within-gender comparisons, i.e. homosexual men vs. heterosexual men and homosexual women vs. heterosexual women (one-sided in both directions). Absolute threshold mask was set to 10% limiting the analysis to voxels with more than 10% probability of containing GM. Resulting statistical parametric maps were corrected for multiple comparisons in the entire volume using the false discovery rate (FDR) method (p<0.05) [25] on the voxel level.

## Results

### Global effects of gender and sexual orientation

Male brains were larger than female brains. Two-way ANOVAs (with gender and sexual orientation as grouping factors) revealed higher mean tissue voxel values of males compared to females in GM (F_1,76_ = 24.8; p<0.001), WM (F_1,76_ = 20; p<0.001) and cerebrospinal fluid (CSF) (F_1,76_ = 23.9; p<0.001). No main effect for sexual orientation and no interaction of the two grouping factors were detected in any tissue type. By means of post hoc t-tests for independent samples mean voxel tissue values were compared between heterosexual and homosexual subjects within each gender group. No differences were found expect for a higher degree of CSF in homosexual men compared to heterosexual men (t_(38)_ = −2.14; p = 0.04; two-sided) (Table 1).

**Table 1:** Mean GM, WM, and CSF volumes (ml) of heterosexual and homosexual men and women.

		GM	WM	CSF	n
Men		858±59	568±43	387±30	40
	heterosexual	856±68	565±47	377±24	24
	homosexual	863±45	572±37	398±33	16
Women		795±52	515±58	345±47	40
	heterosexual	796±53	523±59	346±51	25
	homosexual	794±53	518±60	341±41	15
Total	heterosexual	825±67	538±59	362±43	49
	homosexual	829±59	546±56	370±47	31
	Total	826±64	541±57	365±44	80

Mean volume values as delivered by SPM segmentation algorithm.

GM = grey matter, WM = white matter, CSF = cerebrospinal fluid.

### Regional effects of gender and sexual orientation

There was a significant effect of gender on GM. Brains of (heterosexual and homosexual) women showed areas of increased GM concentration, relatively symmetrically and widespread through the entire cortical mantle, in the cerebellum bilateral and subcortical bilateral in the amygdala, caudate, thalamus, and right hippocampus. Major cortical areas of increased GM concentration could be found in the midline of both hemispheres, spanning from cuneus and posterior cingulate to prefrontal superior gyrus and anterior cingulate as well as bilaterally in the superior parietal lobule. Cohens ‘d effect size of the voxel showing maximum difference between gender (x, y, z = 26, −26, 66) was 1.27.

**Figure 1:**
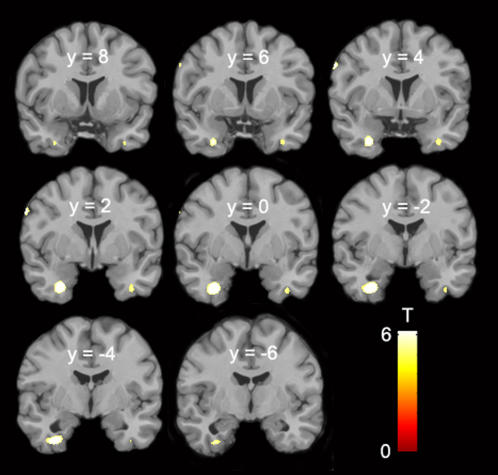
Areas of increased GM concentration in heterosexual women compared to homosexual women. Coronal sections from y = 8 to y = −6 (p<0.05; FDR corrected). Left brain side is to the left.

With regard to within-gender comparison, there were no GM differences between heterosexual and homosexual men, but heterosexual women showed clusters of increased GM concentration in comparison to homosexual women bilaterally in the temporo-basal cortex, ventral cerebellum, and left ventral premotor cortex. Higher GM concentrations were most prominent in the left perirhinal cortex, resembling the larges cluster of increased GM concentration spanning ventrally from the collateral sulcus, to the rhinal sulcus at the ventral border of the entorhinal cortex and extending to the ventral margin of the amygdala (Fig. 1, Table 2).

**Table 2:** Areas of increased GM concentration in heterosexual women compared to homosexual women.

Area	Side	Cluster extent	Peak difference				
			P	T-value	Coordinates		
					*x*	*y*	*z*
Perirhinal cortex	L	1209	0.011	6.20	−32	1	−35
Ventral premotor cortex	L	101	0.015	5.37	−63	3	36
Cerebellum	L	744	0.015	5.37	−10	−40	−55
Cerebellum	R	303	0.031	4.74	26	−50	−59
Perirhinal cortex	R	240	0.036	4.61	34	3	−36

Regional differences in GM concentration are characterized by the cluster extent, stereotactic MNI coordinates and T-value of the voxel showing the peak difference. P-values were corrected by the False Discovery Rate. R = right; L = left.

**Figure 2:**
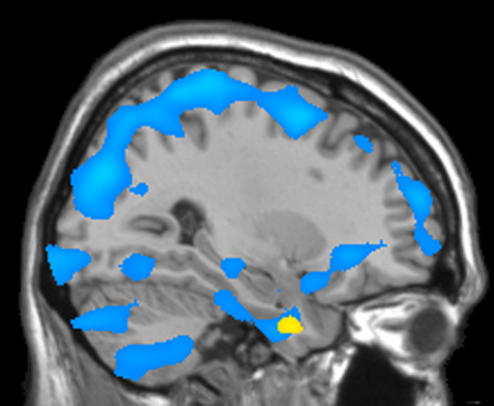
Heterosexual men and homosexual women compared to heterosexual women. Areas of decreased GM concentration in heterosexual men are shown in blue and areas of decreased GM concentration in homosexual women are shown in yellow (p<0.05; FDR corrected, sagittal section at x = −29). Reduced GM concentration of homosexual women (relative to heterosexual women) is located within a sex dimorphic brain area.

Finally, we tested for common areas of decreased GM in homosexual women and heterosexual males (both relative to heterosexual women). Therefore the contrast *women (heterosexual*>*homosexual)* was implicit masked with the contrast *heterosexual (women*>*men)* (both FDR corrected). That way the intersection of significant voxels of both contrasts was gathered. As a result we found one cluster that matches both comparisons. That is, this brain area showed both, a lower GM concentration in heterosexual men compared to heterosexual women and a lower GM concentration in homosexual women relative to heterosexual women. Coordinates of the peak difference of this cluster was identical with the above mentioned unmasked cluster (x, y, z = −32, 1, −35) although the masked cluster was smaller (cluster extent 575 voxel; Z = 4.81; p = 0.023; FDR corrected; Fig. 2).

## Discussion

The novel finding of the present study is that homosexual women have less GM in the temporo-basal cortex, ventral cerebellum and left ventral premotor cortex compared with heterosexual women. No difference in GM was found between male homo- and heterosexuals. Finally, we were able to replicate previous findings regarding the influence of gender on regional grey matter in the adult human brain. Most of these differences are not influenced by sexual orientation.

The main morphometric difference between heterosexual and homosexual women was found in the left perirhinal cortex with a relative reduction in GM in homosexual women. This area was also found to be sex-dimorphic, showing a relative reduction in GM in males. This raises the question whether female homosexuality is associated with a sex atypical differentiation of this brain area. The perirhinal cortex is located close to entorhinal cortex, hippocampus, parahippocampal gyrus and amygdala, and is known to be involved in a variety of functions like olfactory processing, memory encoding and spatial processing. These functions are related to the processing of sexual stimuli as well.

Sex differences of the olfactory system are known at a behavioural level [26], [27], and functional neuronal activity [28] and brain morphology [29] of this area are influenced by sex. In humans, olfactory processing changes with menstrual cycle and modifies sexual attraction [30]–[32]. The olfactory system is able to discriminate pheromone-like compounds according to sexual orientation. The subsequent activation of hypothalamic nuclei was recently shown [33], [34]. The perirhinal cortex is also involved in spatial processing and detection of object identity [35]. Of note, spatial processing was found to be influenced by sexual orientation in females, with homosexual women performing higher than heterosexual women [for review see 36]. Animal research suggests that the perirhinal-entorhinal cortical complex is involved in the identification and spatial localization of social odours which help to distinguish among individual conspecifics [37]. Moreover, the perirhinal-entorhinal cortex was shown to be of relevance to identifying conspecifics in the context of the Coolidge effect in male hamsters [38]. These findings provide converging evidence that the perirhinal-entorhinal cortices help to form neuronal representations of object identity, possibly with links to social bonding and sexual behaviour.

Though we show that the perirhinal cortex is morphologically different in women dependent on their sexual orientation, the functional correlates of the difference in GM concentration remains to be explored. Several questions are raised by our finding and may stimulate future directions of research: First, the contribution of the area around the rhinal sulcus to sexual odour processing, sexual behaviour and pair-bonding in humans remains unknown. Second, it is unknown whether homosexual and heterosexual women show behavioural differences in odour perception. Third, the mechanisms leading to the GM differences in the perirhinal cortex remain to be determined.

In addition to the temporo-basal cortex, the ventral cerebellum and left ventral premotor cortex showed less GM compared to heterosexual women. The detected differences in the cerebellum and premotor cortex are difficult to interpret in the context of this exploratory investigation, as these areas were not sex-dimorphic. The functional significance of this morphometric difference needs to be clarified in future studies.

In the male group, sexual orientation was not associated with regional differences in GM. This indicates that at least at the macroscopic level, the cortex of homosexual men shows no male-to-female shift in GM concentration. Conversely, homosexual women showed in some areas, mostly in the temporo-basal cortex, less GM concentration than heterosexual women, depicting a trend towards a male-like GM pattern. This is of particular interest, because the temporobasal cluster showing a male-like decrease in GM concentration was found in an area that also showed changes related to gender. These findings – a “male-like” GM pattern in homosexual women but no “female-like” pattern in homosexual men - suggest that male and female homosexuality is not analogously manifested at a structural level in the human brain. Accordingly, there was no overall effect of sexual orientation on regional GM when considering women and man together.

The discrepant morphometric changes parallel other findings of sex-dimorphic features that were more male-like in homosexual women, but not female-like in homosexual men, such as the 2D∶4D finger ratio, otoacoustic emissions, and body build [see for review 39]. This adds to suggestions in the literature that sex atypical levels of prenatal androgen action might be involved in the origin of female but not of male homosexuality [39]. However, the more male-like GM concentration in some brain areas in homosexual women is not necessarily a consequence of gender atypical early androgenization. Alternatively, behavioural differences over an individual’s lifetime may account for the morphometric differences between homosexual and heterosexual women.

In agreement with numerous studies [1]–[7], the three tissue compartments of the brain were found to be larger at a global level in males than in females. There were no changes in the absolute volume of the brain tissue compartments which were related to the sexual orientation of the participants. At a regional level, GM concentration was higher in females than in males. Again this finding is in accordance with previous research and resembles the most verified regional sexual brain dimorphism [2], [4], [9], [11], [40]. This indicates that our study sample was sufficient to identify the well-known gender related differences of the human brain. The detected areas of increased GM concentration were distributed about the entire cortical mantle, emphasizing the midline areas of both hemispheres spanning from cuneus and posterior cingulate to prefrontal superior gyrus and anterior cingulate and the superior parietal lobule. Furthermore higher GM concentration was found bilateral in the cerebellum, limbic structures and thalamus.
